# Coupling Genetic and Demographic Data to Reveal Dispersal Processes in Emperor Penguins

**DOI:** 10.1002/ece3.71367

**Published:** 2025-05-14

**Authors:** Jimmy Garnier, Gemma Clucas, Jane Younger, Bilgecan Sen, Christophe Barbraud, Michelle LaRue, Alexander D. Fraser, Sara Labrousse, Stéphanie Jenouvrier

**Affiliations:** ^1^ CNRS, Univ. Grenoble Alpes, Univ. Savoie Mont Blanc, LAMA Chambery France; ^2^ Cornell Lab of Ornithology Cornell University Ithaca New York USA; ^3^ Milner Centre for Evolution University of Bath Bath UK; ^4^ University of Maryland Center for Environmental Science Frostburg Maryland USA; ^5^ Biology Department Woods Hole Oceanographic Institution Woods Hole Massachusetts USA; ^6^ Centre d'Etudes Biologiques de Chizé CNRS‐La Rochelle University UMR7372 Villiers en Bois France; ^7^ School of Earth and Environment University of Canterbury Christchurch New Zealand; ^8^ School of Earth and Environmental Sciences University of Minnesota Minneapolis Minnesota USA; ^9^ Australian Antarctic Program Partnership Institute for Marine and Antarctic Studies, University of Tasmania, Nipaluna Hobart Tasmania Australia; ^10^ Laboratoire d'Océanographie et du Climat: Expérimentations et Approches Numériques (LOCEAN), UMR 7159 Sorbonne‐Université, CNRS, MNHN, IRD Paris France

**Keywords:** dispersal distance, dispersal kernel, dispersal range, emigration rates

## Abstract

Dispersal is a ubiquitous phenomenon that affects the dynamics of the population and the evolution of natural populations; however, it is challenging to measure in most species. Furthermore, the influence of informed dispersal behaviors, referring to the nonrandom selection of breeding habitats by individuals, on species' responses to rapid global change is substantial but difficult to comprehend. Here, we present a modeling framework to assess the dispersal characteristics and behaviors of a metapopulation when observations provide information on its neutral genetic structure for a restricted sampling of locations. Our mechanistic‐statistical model couples a deterministic model capturing the spatio‐temporal dynamics of four genetic clusters across all breeding colonies by integrating demographic processes with genetic projections, with a probabilistic observation model describing the probability to sample an individual from a given genetic cluster. We apply this new framework to the emperor penguin, a species living in Antarctica and currently experiencing habitat loss. The model estimates the species' dispersal distance, rates of emigration, and behaviors associated with dispersal (informed or random). By incorporating these estimations with satellite censuses of breeding colonies, we can identify environmental and demographic factors that influence the dispersal of emperor penguins. Finally, we provide new global population forecasts for emperor penguins that can inform conservation actions in Antarctica.

## Graphical abstract

Dispersal is ubiquitous but dispersal rates, range, and behaviors remain difficult to measure. By developing new models combing demographic and genetic data, we propose to unravel species dispersal behaviors and apply our methodology to the emperor penguins, living in the most remote place on earth where measurements of dispersal are nonexistent. We found that penguins likely depart from their colony when the habitat becomes unsuitable when food resources are low and disperse over short distances relative to their movement capacity.

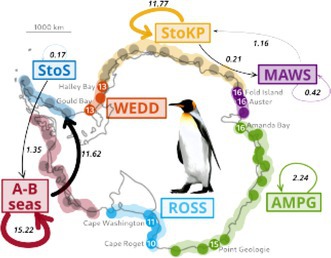

## 1 Introduction

Dispersal between suitable habitats influences the dynamics of populations (e.g., refs (Hastings 1983; Clobert et al. 2004; Bowler and Benton 2005; Levin et al. 2003; Cayuela et al. 2018)), their gene flow and genetic structure (Slatkin 1987; Bohonak 1999; Hewitt 2000; Roques et al. 2012), and hence the ecological and evolutionary processes driving biodiversity (McPeek and Holt 1992; Olivieri and Gouyon 1997; Cadotte 2006; Ronce 2007). The rate and range of dispersal of plant propagules and animal individuals are commonly characterized by tracking individual movements and population redistribution (e.g., using abundance data (Roques et al. 2011) or “mark recapture/sighting” techniques in animal studies (Turchin 1998; Ovaskainen et al. 2008; Southwood and Henderson 2009; Lagrange et al. 2014)). However, such movement data are extremely challenging to collect, especially for endangered species or animals living in remote places on Earth. Genetic markers naturally present in populations offer unique opportunities to study dispersal (Nathan et al. 2003; Hamrick and Trapnell 2011; Robledo‐Arnuncio 2012). However, such genetic methods (e.g., long‐term frequency‐based approach using population structure described by the FST fixation index) estimate effective dispersal over several generations, rather than dispersal processes relevant for the temporal scales at which ecological and demographic processes occur.

Recently, many methods have been developed to assess the dispersal distance kernel over one generation based on genetic data, especially to estimate seed dispersal kernels (Robledo‐Arnuncio and García 2007; Klein et al. 2013; Gelmi‐Candusso et al. 2019). Although these methods are accurate (Jaquiéry et al. 2011), they often rely on simple dispersal assumptions. For example, classical methods based on Euclidean distances or least‐cost distances (e.g., in models of isolation by distance (Wright 1943; Rousset 1997; Broquet et al. 2006)) assume a single and optimal movement path for individuals, while individuals may change their route during dispersal (Stamps 2001; Clobert et al. 2009). Newer methods have been developed that are based on resistance networks (McRae 2006; Graves et al. 2014). These methods consider the relative cost of dispersal in a specific landscape compared to a reference condition. However, their implementation is time‐consuming and the estimation of dispersal parameters, for example, by maximum likelihood, generally lacks accuracy (Graves et al. 2013). Furthermore, genetic data alone may not provide enough information on demographic processes because dispersal processes may depend on the environment (Lowe and Allendorf 2010), the population sizes in different environments may vary, dispersal may occur at short or long distances and dispersal might also depend on individual choice (Jaquiéry et al. 2011).

Here, we integrate genetic methods with environment‐dependent metapopulation models to develop a new likelihood function that quantifies dispersal rates, distances, and behaviors. This novel approach advances previous methods by linking movement and demographic patterns with genetic data (Roques et al. 2016). Specifically, it is based on a mechanistic‐statistical approach (Roques et al. 2011; Ovaskainen et al. 2008; Southwood and Henderson 2009; Berliner 2003; Wikle 2003; Soubeyrand and Roques 2014) in the framework of state‐space models (Patterson et al. 2008; Durbin and Koopman 2012). It has been theoretically developed to characterize insect diffusion rates based on genetic data over a single generation (Roques et al. 2016), but it has yet to be applied to other species. In addition, this method has ignored reproductive and dispersal behaviors. The latter is particularly important, as some species use personal and social information to decide whether to leave a natal or current breeding site and where to settle (e.g., (Doligez et al. 2002)). Such “informed dispersal” behavior (Clobert et al. 2009) enables individuals to settle in habitats of better quality, potentially improving their fitness, therefore increasing population viability and species persistence, especially in the face of global changes (Ponchon et al. 2015).

In this study, we present a likelihood function for a metapopulation mechanistic‐statistical model that integrates reproductive and dispersal behaviors, including informed departure and settlement decisions. We apply this model to emperor penguins ( *Aptenodytes forsteri* ), an Antarctic seabird that is increasingly threatened by climate change (Jenouvrier et al. 2021). Due to the logistical challenges of monitoring populations in extreme environmental conditions, very little is known about their dispersal behaviors. In fact, emperor penguins have only been marked at one site (Pointe Géologie (Barbraud and Weimerskirch 2001)), with no recaptures elsewhere. The recent advent of satellite telemetry tags has allowed for an enhanced understanding of the movement of emperor penguins on large spatial scales within a season. However, this approach is not suitable for determining dispersal between colonies due to the limited life span of these devices (Thiebot et al. 2013).

Like many seabirds, emperor penguins are considered highly philopatric (Mougin and Van Beveren 1979). However, this traditional view has been challenged by advances in genetic analyses and very high‐resolution satellite imagery (VHR), suggesting that movements between colonies occur (LaRue et al. 2015). In fact, genetic studies have identified at least four distinct genetic clusters among emperor penguins (Younger et al. 2017). Each cluster is located in a different geographic region of Antarctica, some spanning thousands of kilometers of coastline and comprising multiple breeding colonies. While there is some degree of gene flow connecting these clusters, they remain genetically distinct from one another. However, within each cluster, the dispersal of individuals between breeding colonies is sufficient to maintain panmixia (Younger et al. 2017). In addition, VHR satellite imagery has recently documented colony movements, disappearances, and relocations (Fretwell and Trathan 2021). For example, a dramatic decline in the world's second‐largest emperor penguin colony occurred at Halley Bay, while the nearby Dawson‐Lambton colony, 55 km to the south, saw a more than tenfold increase in penguin numbers during the same period (Fretwell and Trathan 2019). Halley Bay has suffered 3 years of almost complete breeding failure caused by a change in the local environment and sea ice conditions, and those unfavorable conditions may have forced penguins to relocate to Dawson‐Lambton (Fretwell and Trathan 2019). The colony had been present at Halley Bay since at least 1956, persisting for 60 years before the major environmental disturbance led to a massive population decline and emigration event. This suggests that emperor penguin movements may be triggered by major environmental disturbances and that individuals leave their current breeding site using information about their habitat quality, such as the presence of a stable and suitable ice habitat to breed. These dispersal behaviors correspond to informed emigration.

Using a mechanistic‐statistical approach, we have developed a likelihood function that links the demographic characteristics of the emperor penguin to genetic data. It enables us to: (1) determine the most likely dispersal behaviors in emigration and establishment of emperor penguins (informed vs. random); (2) estimate the mean dispersal distance for this species and the emigration rates between colonies; (3) highlight environmental and demographic factors that drive emigration rates by combining our approach with independent demographic and environmental data; and (4) project the global population of emperor penguin change into the future using the most recent large ensemble of climatic projection in Antarctica (CESM2‐LENS (Rodgers et al. 2021)).

## 2 Material and Methods

The model presented in this study is based on the combination of a mechanistic metapopulation model that describes the population dynamics, and a stochastic model that accounts for the collection of genetic measurements based on the population dynamics (section “Mechanistic‐statistical model”) (Soubeyrand et al. 2009). A previous theoretical study has demonstrated the relevance of this approach for estimating dispersal parameters using genetic data (Roques et al. 2016). We begin by describing our case study, the emperor penguin (section “Case study: emperor penguin”). Then we present the available genetic data for emperor penguins (section “Genetic data”), followed by an explanation of our mechanistic‐statistical approach and the statistical inference of the model parameters (section “Statistical inference”). Finally, we compare the different dispersal behavior (section “Comparison of dispersal behaviors”), we evaluate the impact of demographic and environmental factors on emigration rates (section “Impact of demographic and environmental factors on emigration rates”) and project the future dynamics of the global emperor penguin population until 2100 (section “Forecasts of emperor penguin global population”).

### 2.1 Case Study: Emperor Penguin

**FIGURE 1:**
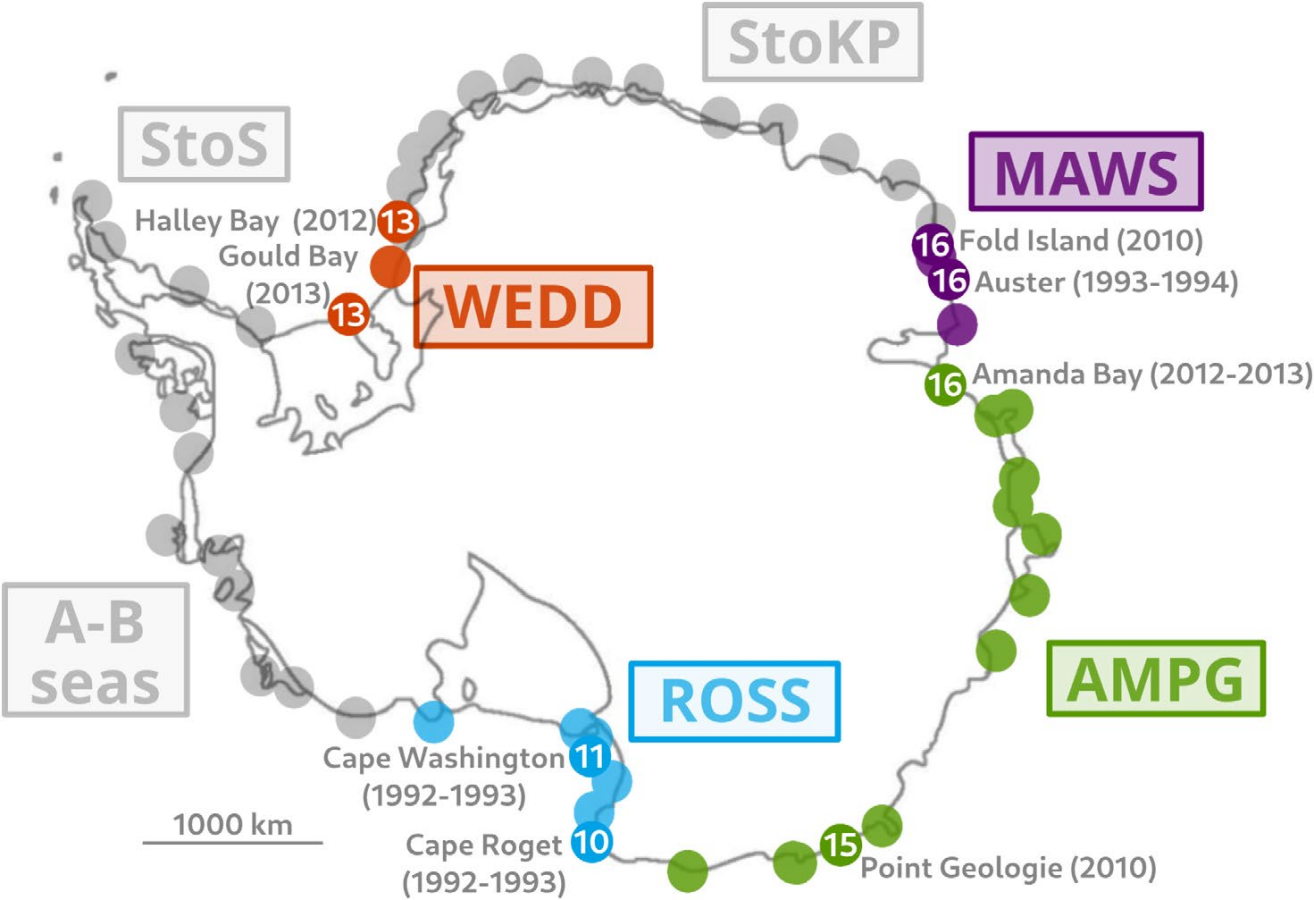
The four genetic clusters detected around Antarctica that characterize four geographic regions: (WEDD) Weddell Sea (Gould Bay to Halley Bay colonies) red dots, (MAWS) Mawson Bay (Fold Island to Cape Darnley colonies) purple dots, (AMPG) Amanda Bay to Pointe Geologie colonies, green dots and (ROSS) Ross Sea (Cape Washington and Cape Crozier colonies) blue dots. Gray and colored dots indicate all the 66 known emperor penguins colonies around Antarctica. The three gray regions corresponds to are without genetic characterization: (StoS) from Smith to Snowhill Island in the Weddell sea colonies, (StoK) from Stancomb to Kloa point colonies and (A–B Seas) Ledda bay to Rotschild colonies (Admunsen and Bellingshausen Seas). The white numbers indicated the number of individuals sampled from this colonies and the year of sample.

Emperor penguins are seabirds that live in Antarctica. They breed annually during the Antarctic winter in one of the 66 breeding colonies around Antarctica (see circles in Figure 1). In March, adults settle in a colony to mate, lay a single egg, and raise their chick until December. Adults and juveniles leave colonies in December/January and disperse into the Southern Ocean. After a northward migration following departure from their natal colony, juveniles return close to the Antarctic sea ice in April/May (see (Prevost 1961) and observations from Argos tracking (Labrousse et al. 2019; Thiebot et al. 2013)). The first breeding starts at 3 years. During this period, from fledgling to first breeding, individuals can prospect (Mougin and Van Beveren 1979; Prevost 1961) and eventually assess the habitat quality of a potential colony to settle and breed.

A recent genetic study identified four genetic clusters among emperor penguins that are significantly genetically differentiated, with some degree of gene flow connecting these clusters (Younger et al. 2017). Although genetic clustering (STRUCTURE) (Pritchard et al. 2000) and FST analyses support the presence of four genetic clusters, genetic differentiation between emperor penguin colonies is subtle and hierarchical, and does not follow a typical isolation‐by‐distance (IBD) pattern of differentiation.

The four identified genetic clusters coincide with distinct geographical regions composed of several colonies: (WEDD) Weddell sea (Gould Bay to Halley Bay colonies), (MAWS) Mawson Bay (Fold Island to Cape Darnley colonies), (AMPG) Amanda Bay to Pointe Geologie colonies, and (ROSS) Ross sea (Cape Washington and Cape Crozier colonies) (see Figure 1). However, these four geographical regions do not cover all the 66 colonies around Antarctica. Three geographical regions remain genetically uncharacterized:(StoS) from Smith to Snowhill Island in the Weddell sea colonies, (StoK) from Stancomb to Kloa point colonies, and (A‐B seas) Ledda bay to Rotschild colonies (Admunsen and Bellingshausen seas) (sees Figure 1). Although it is possible that there are more than four genetic clusters across the emperor penguin's range, a complete sampling of all colonies is logistically infeasible due to the remote distribution of the species in one of the harshest climates on Earth. This limitation is common in wildlife studies, where representative sampling is often the only viable option. Our estimations of dispersal parameters are therefore conditional on the assumption that the emperor penguin population is structured into these four identified genetic clusters.

### 2.2 Genetic Data

We used genetic data collected from 1992 to 2013 in eight colonies around Antarctica by (Younger et al. 2017) (see Figure 1). After filtering steps (Benestan et al. 2016), 4.596 neutral genome‐wide single nucleotide polymorphisms (SNPs) were retained. Specifically, we here use only SNPs that are present in all sampled colonies (parameter‐p 8 in the Stacks pipeline) and that are present in at least 80% of individuals per colony (parameter‐r 0.8 in the Stacks pipeline). We keep SNPs that are neutral and polymorphic at the metapopulation level. However, some allele frequencies in genetic clusters might be equal to 0 because some alleles are private in the sense that they appear only in one genetic cluster. A total of 110 individuals (10–16 per colony) were successfully genotyped at these loci.

### 2.3 Mechanistic‐Statistical Model

The mechanistic model characterizes the spatio‐temporal changes of emperor penguin populations across the 66 colonies in Antarctica. It combines a demographic model, describing the metapopulation dynamics and incorporating various parameters such as mean dispersal distance, emigration rates, and dispersal behaviors (section “Demographic model”), with a genetic population model projecting the number of individuals originating from one of the four genetic clusters for each year and in each colony (section “Genetic population dynamics”). The stochastic model, on the other hand, includes a probabilistic sampling approach to estimate the likelihood of sampling an individual from a given genetic cluster (section “Probabilistic sampling model”) and a statistical genetic model that predicts the probability of observing a particular genotype based on the individual's genetic cluster of origin.

#### 2.3.1 Demographic Model

We use the metapopulation model developed by (Jenouvrier et al. 2017) to project the female population vector n, that comprises the female population size $n_{i}$ in each colony i, from year t to year t+1 (we only look at the female here):

(1) $$n\left(t+1\right)=D\left[t,n\left(t\right)\right]F\left[t,n\left(t\right)\right]\;n\left(t\right)$$

It incorporates two phases of possibly different duration: a motionless density‐dependent reproduction phase (F) followed by a dispersal phase including natal or breeding dispersal (D). The reproduction matrix F follows a Ricker model, where the intrinsic growth rate $r_{i}\left(t\right)$ of each colony varies in time due to sea ice concentration (SIC) variations, described by climatic projection in Antarctica (CESM2‐LENS (Rodgers et al. 2021)), while carrying capacities of colonies are constant over time. The dispersal phase D comprises three stages: (1) emigrating from the resident colony at a rate $m_{i}\left(t\right)$, (2) searching for a new colony among other colonies with an average dispersal distance d (transfer), and (3) settling in a new colony. During the emigration and settling stages, two possible behaviors (informed versus random) can occur:

- An informed emigration: individuals only leave poor‐quality breeding sites if habitat quality is no longer viable, that is, when population growth rate in the site $r_{i}$ is negative, $r_{i}\left(t\right)<0$. In this case, emigration occurs at a rate $m_{i}\left(t\right)=\max\left(1-r_{i}\left(t\right)/\left(\left(1-p_{m_{i}}\right)r_{c}^{*}\right),1\right)$, which depends on the current habitat quality relative to the worst possible quality. This is measured through the population growth rate ratio $r_{i}\left(t\right)/r_{c}^{*}$, where $r_{c}^{*}<0$ represents the lowest growth rate. Additionally, the parameters $p_{m}=\left(p_{m1},\ldots ,p_{m7}\right)$ quantify the sensitivity to poor‐quality habitat and determine the intensity of emigration in each region. A value of $p_{m}$ close to 1 implies that even minor habitat degradation triggers a sharp increase in emigration rate, while lower values of $p_{m}$ lead to a more gradual response.
- A random emigration: individuals leave the colony regardless of the habitat quality at a fixed rate $m_{i}=p_{m_{i}}$. In this context, the parameter $p_{m_{i}}$ represents the proportion of individuals that leave the colonies in region i;
- An informed establishment: individuals select the most suitable habitats (i.e., maximize intrinsic population growth) within their dispersal range of size d;
- Random establishment: individuals pick a colony in their dispersal range of size d randomly and regardless of the habitat quality of the colonies.

In our analysis, we only consider three dispersal behaviors: the random dispersal behavior (R) with random emigration and establishment; the semi‐informed dispersal behavior (SI) with informed emigration but random establishment; and the informed dispersal behavior (I) with both informed emigration and establishment.

#### 2.3.2 Genetic Population Dynamics

The population of emperor penguin comprises 4 genetic clusters characterized by their allele frequencies $F_{r\lambda }={\left(p_{r\lambda a}\right)}_{a=1,\overset{\ldots }{,}A_{\lambda }}$ at the sampled SNPs λ (with possibly some allele frequencies equal to 0 due to the presence of private alleles in some genetic clusters). The frequencies are assumed constant over the sampling interval because they represent less than two generations for emperor penguins (which is 16 years) (Jenouvrier et al. 2014). Our demographic model describes the survival, reproduction and dispersal of individuals regardless of their native genetic cluster. In order to track the native genetic cluster of individuals, we derive from the demographic model (Roques et al. 2012, 2016), a genetic model that projects the population vector $n^{r}$ of individuals that originate from one of the four genetic cluster r. The vector $n^{r}$ comprises the number of individuals $n_{\tau }^{r}\left(t\right)$ in each colony τ originating from cluster r, and satisfies the following dynamics

$$n^{r}\left(t+1\right)=D\left[t,n\left(t\right)\right]F\left[t,n\left(t\right)\right]\;n^{r}\left(t\right).$$

Initially, we have the following repartition

(2) $$n_{i}^{r}\left(0\right)=\mu_{i}^{r}n_{i}\left(0\right),\;\text{for}\;\mathrm{all}\;i\in \left\{1,\ldots ,66\right\},$$

where $\mu_{i}^{r}$ is the initial proportion of individuals native from genetic cluster r within the colony i ($\sum_{r=1}^{R}\mu_{i}^{r}=1$ for all i). We assume that for colonies belonging to a region where we have genetic information, the proportion is 1 if the geographical region of the colony matches the genetic cluster and 0 otherwise. For instance, if the colony i belongs to the region that matches genetic cluster 1, then

$$\mu_{i}^{1}=1\;\text{and}\;\mu_{i}^{2}=\mu_{i}^{3}=\mu_{i}^{4}=0.$$

However, for the colonies belonging to the three geographical regions without genetic information, the parameters $\mu =\left(\mu_{i}^{1},\mu_{i}^{2},\mu_{i}^{3},\mu_{i}^{4}\right)$ are unknown parameters that we have to estimate. In order to simplify the estimate, we assume that μ is the same among colonies of a geographical region. The genetic and demographic dynamics are linked by:

$$n_{\tau }\left(t\right)=\sum_{r=1}^{4}n_{\tau }^{r}\left(t\right).$$

More precisely, since the set of SNPs in our genetic data is selectively neutral, individuals within a colony share the same dispersal and reproduction characteristics independent of their genetic background. In particular, an individual within a colony τ and originating from the genetic cluster r produces offspring at the same rate as any individual within the colony τ. Although newborns result from the mating of a male and a female, we assume that they inherit the native genetic cluster of the female.

#### 2.3.3 Probabilistic Sampling Model Associated With the Demographic Model

During the year t that ranges from 1992 to 2013, individuals from colonies $J_{t}$ were genotyped. The sampling of individuals in a given year t, in the colony τ is random among the individuals observed at the colony. Given that our dataset has an average sample size per colony $G_{\tau }$ that is relatively small (10–16 individuals per colony) in comparison to the typical population size of emperor penguin colonies (which ranged from 100 to 25,000 individuals in 2009 (Fretwell and Trathan 2009)), the count of genotyped individuals in τ originating from genetic cluster r follows a multinomial distribution characterized by the parameters $G_{\tau }$, the sample size, and $\left(\mu_{\tau }^{1}\left(t\right),\ldots ,\mu_{\tau }^{R}\left(t\right)\right)$, the proportions of individuals in colony τ at time t, that originate from one of the four genetic clusters. The probability that a genotyped individual i observed at time t in colony τ, originates from genetic cluster r, is

$$P\left(\;\text{indiv}.\;i\;\text{originates}\;\text{from}\;\text{cluster}\;r\;\right)=\mu_{\tau }^{r}\left(t\right)=\frac{n_{\tau }^{r}\left(t\right)}{n_{\tau }\left(t\right)}.$$

The proportion $\mu_{\tau }^{r}$ corresponds to the ratio between the number of individuals $n_{\tau }^{r}\left(t\right)$, that originate from the genetic cluster r and the number $n_{\tau }\left(t\right)$ of individuals alive in the colony τ projected by the metapopulation model.

#### 2.3.4 Statistical Genetic Assignment Approach

Emperor penguins are diploid organisms, thus their genotypes write $G={\left\{\left(a_{\lambda }^{1},a_{\lambda }^{2}\right)\right\}}_{\lambda =1,\overset{\ldots }{,}\Lambda }$. As we use single‐nucleotide polymorphisms (SNPs), each locus has two alleles, corresponding to the two possible nucleotide variations in the DNA sequence. Using the linkage equilibrium among loci and the Hardy–Weinberg equilibrium assumption within a genetic cluster, the conditional probability for the genotype $G_{\mathrm{i\tau}}$ is:

$$P\left(G_{i,\tau }|\;\text{indiv}.\;i\;\text{originates}\;\text{from}\;r\;\right)=2^{k_{i}}\prod_{\lambda =1}^{\Lambda }p_{\mathrm{r\lambda}a^{1}}p_{\mathrm{r\lambda}a^{2}}$$

where $k_{i}$ is the number of heterozygous loci in $G_{i,\tau }$, $p_{\mathrm{r\lambda}a^{1}}$ and $p_{\mathrm{r\lambda}a^{2}}$ are the allele frequencies within the genetic cluster r of the alleles $a^{1}$ and $a^{2}$ of individual i at locus λ.

### 2.4 Statistical Inference

#### 2.4.1 Computation of the Likelihood Function

The unknown parameters Θ of our mechanistic‐statistical model are the mean dispersal distance d, the emigration sensitivity parameters $p_{m}=\left(p_{m_{1}},\ldots ,p_{m_{7}}\right)$ and the initial proportions of each genetic clusters, $\mu ={\left(\mu_{i}^{1},\mu_{i}^{2},\mu_{i}^{3},\mu_{i}^{4}\right)}_{i\in \left\{1,2,3\right\}}$. For the three different dispersal behaviors (random, semi‐informed or informed dispersal) and with unknown parameters Θ of our model, the likelihood function is:

$$L\left(\Theta |\;G_{i,\tau }\right)=\prod_{t=1992}^{2013}\prod_{\tau =1}^{J_{t}}\prod_{i=1}^{G_{\tau }}P\left(G_{i,\tau }\right)$$

where $P\left(G_{i,\tau }\right)$ is the probability to sample the genotype $G_{i,\tau }$ in colony τ at time $t_{i}$, that can be decomposed as follows

$$\begin{array}{l} P\left(G_{i,\tau }\right)=\sum_{r=1}^{4}\;P\left(G_{i,\tau }|\;\text{indiv}.\;i\;\text{originates}\;\text{from}\;\text{cluster}\;r\;\right)\\ \;\;\;\;\;\;\;\;\;\;\;\;\;\;\;\;\;\;\;\;\;\times \;P\left(\;\text{indiv}.\;i\;\text{originates}\;\text{from}\;\text{cluster}\;r\;\right) \end{array}$$

For each dispersal behavior (random (R), semi‐informed (SI) or informed (I)), we estimate the posterior distribution of the parameters Θ using the likelihood function and an importance sampling algorithm with prior distribution of parameters d and $p_{m}$ given by uniform distribution with the following constraints:

$$\left(d,p_{m}\right)\in \left(250,6500\right)\times {\left(0,1\right)}^{7}$$

and prior of the parameter μ given by a Dirichlet distribution of order R=4 with parameters all equal to 1:

$$\mu_{h}^{r}\in {\left(0,1\right)}^{4}\;\text{and}\;\sum_{r=1}^{4}\mu_{h}^{r}=1\;\text{for}\;\mathrm{all}\;h\in \left\{1,\ldots ,66\right\}.$$

We performed the statistical inference with Matlab version R2021a. The code is available online at https://github.com/garnieji/EP_demographic_genetic.

#### 2.4.2 Confidence Intervals and Goodness‐Of‐Fit

The model's goodness‐of‐fit was evaluated by determining the 95% confidence regions for the observed genotypes in each colony and year of observation. To do this, we calculated the probability of each possible observed genotype based on the frequencies of each genetic cluster predicted by the mechanistic model using the estimated parameters that maximize the likelihood function. We then checked if the observed genotypes fell within the 95% confidence regions that represent the most likely outcomes.

### 2.5 Comparison of Dispersal Behaviors

In order to determine the most probable dispersal behavior, we performed a model selection process using the Deviance Information Criterion (DIC), defined as:

(3) $$\mathrm{DIC}=\hat{D}+\frac{1}{2}V\left(D\left(\Theta \right)\right)$$

where $\hat{D}$ and $V\left(D\left(\Theta \right)\right)$ are, respectively, the posterior mean and variance of the deviance, given by $D\left(\Theta \right)=-2\;\log\left[L\left(\Theta \right)\right]$. Minimizing the posterior mean ensures a good fit, while the posterior variance reflects the effective number of parameters in the model (Gelman et al. 2003). Although this criterion produces results similar to the Bayesian Information Criterion (BIC), which penalizes the maximum likelihood estimate based on the number of parameters, the Bayesian nature of the DIC criterion accounts for parameter uncertainty and correlation when sampling from the joint posterior distribution (Ward 2008). In practice, the posterior mean and variance are estimated using their empirical values, computed from the weighted posterior sample $\left\{\Theta_{m},w_{m}\right\}$ obtained through our minimization algorithm.

### 2.6 Impact of Demographic and Environmental Factors on Emigration Rates

First, using the posterior distribution of emigration rates obtained from our mechanistic‐statistical model, we classified colonies into two categories each year: “No emigration” colonies, where the emigration rate is zero, and “Emigration” colonies, where the emigration rate is positive.

Next, to evaluate the influence of demographic and environmental factors on emigration, we characterized emigration using two distinct metrics: (1) Annual emigration probability—the probability that a colony falls into the “Emigration” category in a given year, and (2) Average emigration probability—the proportion of years between 2009 and 2013 in which a colony was classified as “Emigration.” These two metrics offer insight into the propensity of individuals to leave their colony, respectively, in a given year or over a 5‐year period.

Next, we examined various environmental and demographic factors that were independent of our metapopulation model and correlated to habitat quality of colonies which we assume strongly correlated to emigration rate. Environmental factors included zooplankton biomass (mmol $C/m^{2}$) (Offredo and Ridoux 1986; Kirkwood and Robertson 1997; Cherel and Kooyman 1998) and the distance between the colony and the nearest edge of landfast sea ice (m) (Labrousse et al. 2023; Massom et al. 2009). The diet of emperor penguins varies by location and by season, but mainly consists of Antarctic krill *(Euphausia superba)*, various species of fishes (Antarctic silverfish, *Pleuragramma antarctica*, Trematomus species, *Pagothenia borchgrevinki*, and *Pleuragramma antarcticum* ), glacier squids *(Psychroteuthis glacialis)* and Antarctic neosquid *(Alluroteuthis antarcticus)* (Offredo and Ridoux 1986; Kirkwood and Robertson 1997; Cherel and Kooyman 1998). Although Antarctic zooplankton comprises many species, its local biomass could provide insight into the available resources for emperor penguins. As emperor penguins incubate and rear their chicks on landfast sea ice, its extent and the timing of sea ice breakup influence breeding success and, consequently, the suitability of breeding sites (Labrousse et al. 2023; Massom et al. 2009).

Environmental data were obtained from novel landfast sea ice datasets (hereafter referred to as “fast ice”) at different scales (Fraser et al. 2021) and from unique sea ice and food web dynamics variables derived from a forced ocean‐sea ice (FOSI) configuration of the Community Earth System Model (CESM2) (Long et al. 2021). When applicable, we computed the average value of each environmental variable across different breeding periods: non‐breeding (January to March), laying (April and May), incubation (June and July), and chick‐rearing (August to December).

Additionally, we considered three demographic factors: colony size, growth rate per colony, and blinking frequency. From 2009 to 2018, the size and growth rate of each colony were computed annually based on population counts obtained from VHR satellite imagery (LaRue et al. 2022, 2024). The colony size and growth rate per colony were defined as their averages over the 10‐year period (2009–2018). Blinking frequency, derived from colony presence or absence data obtained via VHR satellite imagery, was defined as the proportion of years a colony was absent during the 2009–2018 period (LaRue et al. 2022, 2024).

We employed a random forest algorithm to assess the influence of environmental and demographic variables on emigration (Strobl et al. 2007). Specifically, we used the R package “party” to fit conditional random forests (Strobl et al. 2007), while the “permimp” package (Debeer and Strobl 2020) computed variable importance scores. The corresponding codes are available online at https://github.com/bilgecansen/Emperor_dispersal.

In analyzing annual emigration probability, we considered only the environmental variables around each colony. In contrast, when analyzing average emigration probability, we incorporated both environmental variables (averaged between 2009 and 2013) and the three demographic factors.

### 2.7 Forecasts of Emperor Penguin Global Population

Coupling our new estimated dispersal parameters with the meta–population model developed by (Jenouvrier et al. 2021), we project the total population size of emperor penguins over the century for different climate scenarios. Previous studies have provided a more detailed description of this forecasting approach, which yields a robust forecast by incorporating various sources of uncertainties ((Jenouvrier et al. 2021, 2014, 2009, 2017) for emperor penguins and (DuVivier et al. 2024; Iles and Jenouvrier 2019) for a general approach).

Climate scenarios, which are labeled based on the projected global warming increase (°C) above preindustrial levels, are discussed in greater depth in (Jenouvrier et al. 2020). These scenarios include an increase of 4.3°C [RCP8.5], 2.6°C [new scenario], 2.4°C [RCP4.5], 2°C [Paris 2°C] and 1.5°C [Paris 1.5°C]. The new scenario developed by (Jenouvrier et al. 2020) is intended to demonstrate probable effects on sea ice and therefore emperor penguins by 2100 if governments act now to control greenhouse gas emissions by 2050.

We compare the result of this updated model (semi‐informed dispersal) with the projections of the model without dispersal (see Figure 5a). Finally, we compare projections for different dispersal behaviors and between climate scenarios (see Figure 5b,c).

## 3 Results

### 3.1 Goodness‐of‐Fit and Convergence

From the three dispersal behaviors (random (R), semi‐informed (SI), and informed (I)), we obtained three different set of parameters that maximize the likelihood function. On average, 99% of the observed genotypes fell within the 95% confidence regions, indicating that models with different dispersal behaviors accurately represented the data (109 of the observed genotypes over a total of 110). The three scenarios exhibited a peak in likelihood at the mean dispersal distance parameters, suggesting that these parameters optimize the likelihood for all three scenarios. With respect to the remaining parameters, that are the emigration sensitivity parameters $p_{m_{i}}$ and the initial genetic cluster proportions $\mu_{i}^{r}$, the likelihood function exhibits a smoother distribution around the optimized parameters, indicating that the likelihood is less responsive to changes in these parameters.

### 3.2 Dispersal Processes

#### 3.2.1 Dispersal Behaviors

Based on our model selection, it is evident that the genetic data strongly suggest the prevalence of semi‐informed dispersal behavior among emperor penguins (see Table 1). This behavior indicates that these penguins are more likely to leave colonies with unfavorable habitat conditions (i.e., with negative intrinsic population growth; informed emigration), but settle randomly into another colony (random establishment).

**TABLE 1:** Model selection based on minimization of the deviance information criteria (DIC) defined by (3) for the three different dispersal behavior: random dispersal (random emigration and establishment), semi‐informed dispersal (informed emigration but random establishment) and informed dispersal (informed emigration and establishment).

Dispersal behavior	Random	Semi‐informed	Informed
DIC criteria (Gelman et al. 2003)	684	−41	676

#### 3.2.2 Dispersal Ranges

**FIGURE 2:**
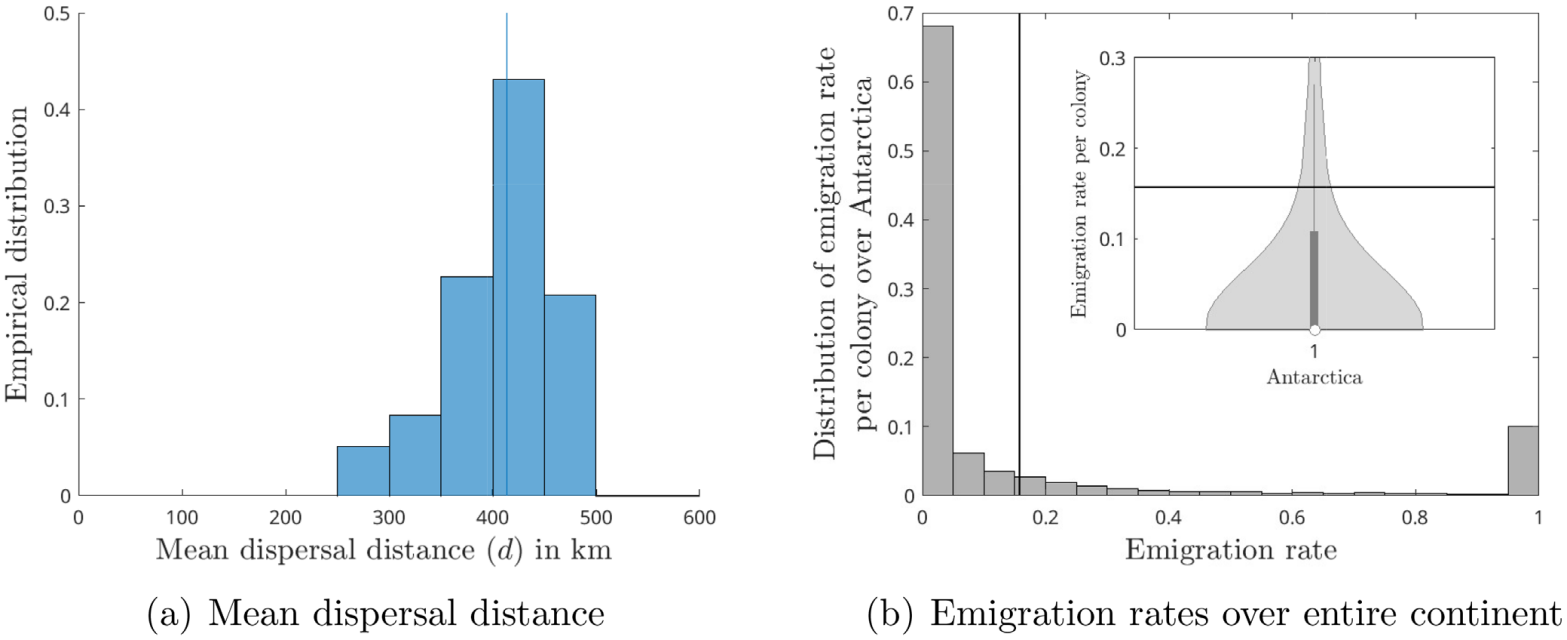
Posterior distributions of the mean dispersal distance d per individuals and the emigration rate per colony per year for the entire Antarctic continent. Plain lines are the mean of the distributions: (a) 414 km (blue) and (b) 0.157 (black).

Figure 2a Shows the posterior distribution of the mean dispersal distance for the best supported model. It indicates a relatively short dispersal distance of approximately 414 km. This mean dispersal distance is modest compared to the potential movement range derived from the tracking of juveniles and adults at sea (tracking studies report traveling distance from 2000 km to 7000, with extreme distance of 9000 km) (Thiebot et al. 2013; Goetz et al. 2018; Kooyman et al. 2004).

#### 3.2.3 Dispersal Rates

**FIGURE 3:**
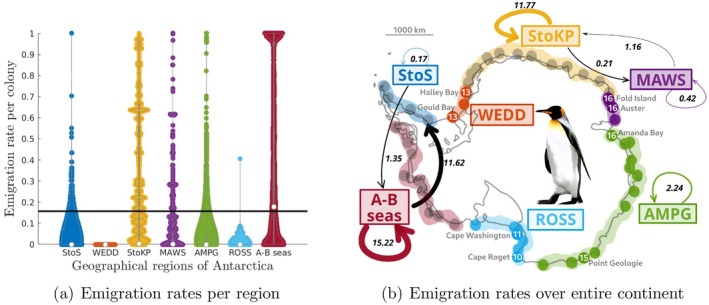
Emigration rates per year per colony (panel (a)) and between and among the seven regions of Antarctica (panel (b)), from 2009 to 2014: From Smith to Snowhill Island in the Weddell Sea (StoS), Weddell Sea (Gould Bay to Halley Bay colonies) (WEDD), from Stancomb to Kloa point (StoK), Mawson Bay (Fold Island to Cape Darnley colonies) (MAWS), from Amanda Bay to Pointe Geologie colonies (AMPG), the Ross Sea (Cape Washington and Cape Crozier) (ROSS) and Admunsen and Bellingshausen seas (Ledda bay to Rothschild Island) (A–B seas). In panel (a), white dots correspond to the median of the posterior distributions of the emigration rates per colony for each region, and the black line is the mean emigration rate for the entire Antarctic continent (0.157). In panel (b), the dots correspond to the 66 colonies of emperor penguins around Antarctica and the color shading indicate their geographical region. Gray dots indicate colonies without genetic information, while colored dots corresponds to the four genetic clusters detected by (Younger et al. 2017). The white numbers indicate the number of individuals sampled in colonies included in our study.

Figure 2b Summarizes the predicted emigration rates across the continent. Dispersal events are generally rare, as indicated by a median emigration rate of zero, meaning that more than 50% of the calculated emigration rates are zero. However, some regions may experience massive emigration events, with emigration rates rising significantly above the average rate, which remains non‐negligible at 15.7% per year (see Figures 2b and 3a).

Figure 3a shows the predicted rate of emigration per colony, organized into clusters based on regions. Furthermore, Figure 3b provides an overview of the average predicted emigration rate both within and between these regions. Emperor penguins move mainly to nearby colonies in the same regions with an average rate per year that varies between regions: 15% in colonies of the Amundsen and Bellingshausen seas (A‐B seas) to 0.17% in colonies from Smith to Snowhill Island in the Weddell sea regions (StoS). However, massive emigration is also likely to occur between different regions, especially between the A‐B seas regions and the StoS regions (11%–1.35%) and colonies from Stancomb to Kloa Point (StoKP) and Mawson bay (MAWS) (1.16%–0.21%).

### 3.3 Potential Drivers of Dispersion

#### 3.3.1 Average Rate of Emigration

**FIGURE 4:**
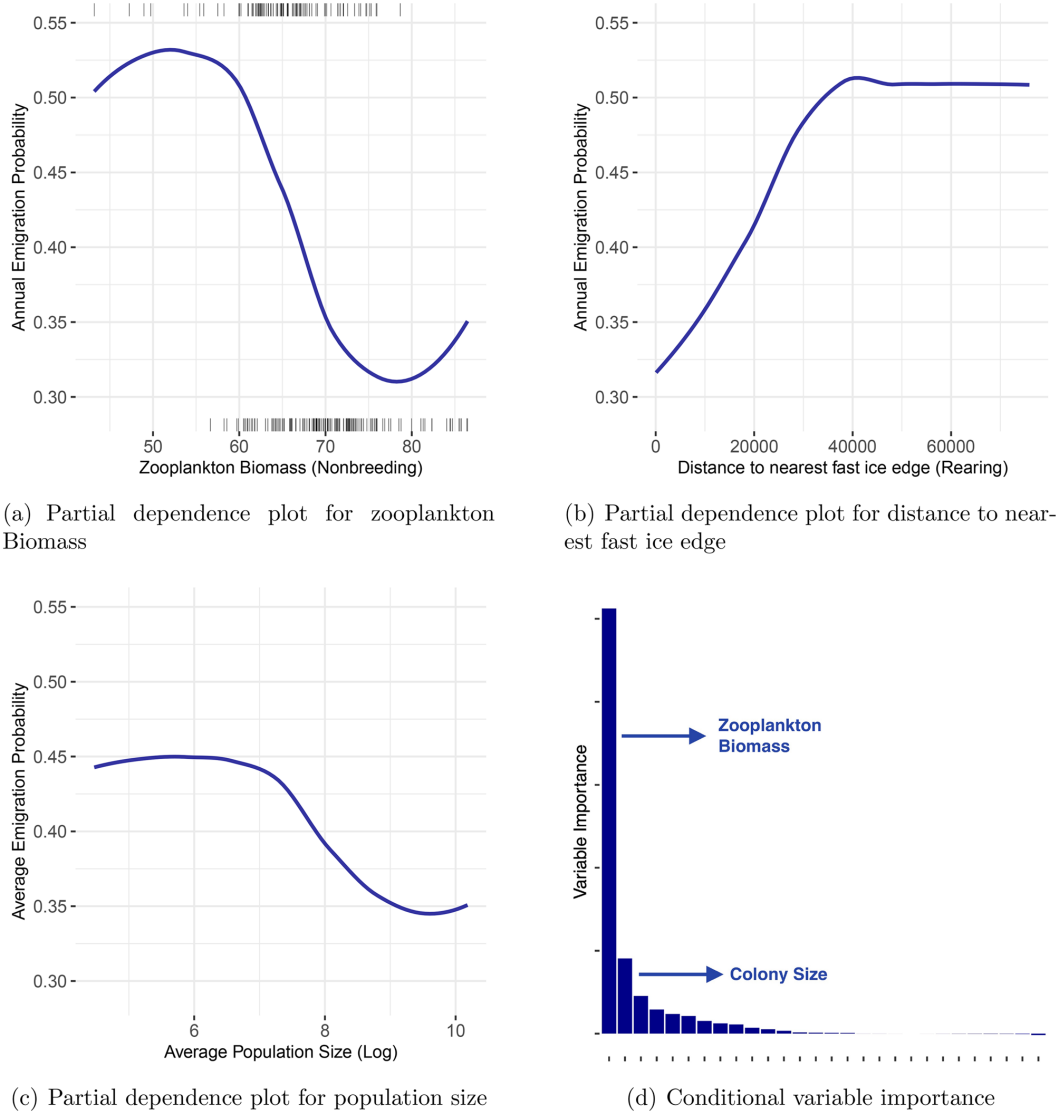
Impact of environmental and demographic factors on emigration rates of emperor penguins: (a) Zooplankton biomass (mmol $C/m^{2}$) during the non‐breeding period (January to March) have a negative effect on annual emigration probability (probability of observing a nonzero median emigration rate in a given year and colony); (b) Distance to nearest fast ice edge (meters) has a positive effect. Lines at the top and bottom of the graphs show the presence (median emigration rate > 0) and absence (median emigration rate = 0) of emigration, respectively, in a given year and colony; (c) the colony size has a negative effect on the average probability of emigration (proportion of years with non‐zero median emigration rates between 2009 and 2013 in a colony); (d) Conditional variable importance scores of random forests modeling average emigration probability. Only the top two variables are shown.

The zooplankton biomass in the non‐breeding season (January to March) stands out as the primary factor influencing the average probability of emigration (the proportion of years with non‐zero median emigration rates between 2009 and 2013 in a colony) among all the evaluated environmental and demographic factors (see Figure 4d). Subsequently, the size of the colony was identified as the second most important factor influencing the average probability of emigration (Figure 4d). Both factors have a negative impact on the rate of emigration (Figure 4a,c). Despite considerable uncertainties, colonies without emigration were found to have an average size almost twice as large as colonies with emigration (3741 penguins compared to 1880 penguins for emigration).

#### 3.3.2 Annual Emigration Probability

Next, we refine our analysis by focusing solely on the annual probability of emigration (the probability of observing a non‐zero median emigration rate in a given year and colony). Our findings indicate that proximity to the nearest fast ice edge positively affects the annual probability of emigration (refer to Figure 4b), ranking second in importance after zooplankton biomass.

### 3.4 Forecasts of Emperor Penguin Global Population

**FIGURE 5:**
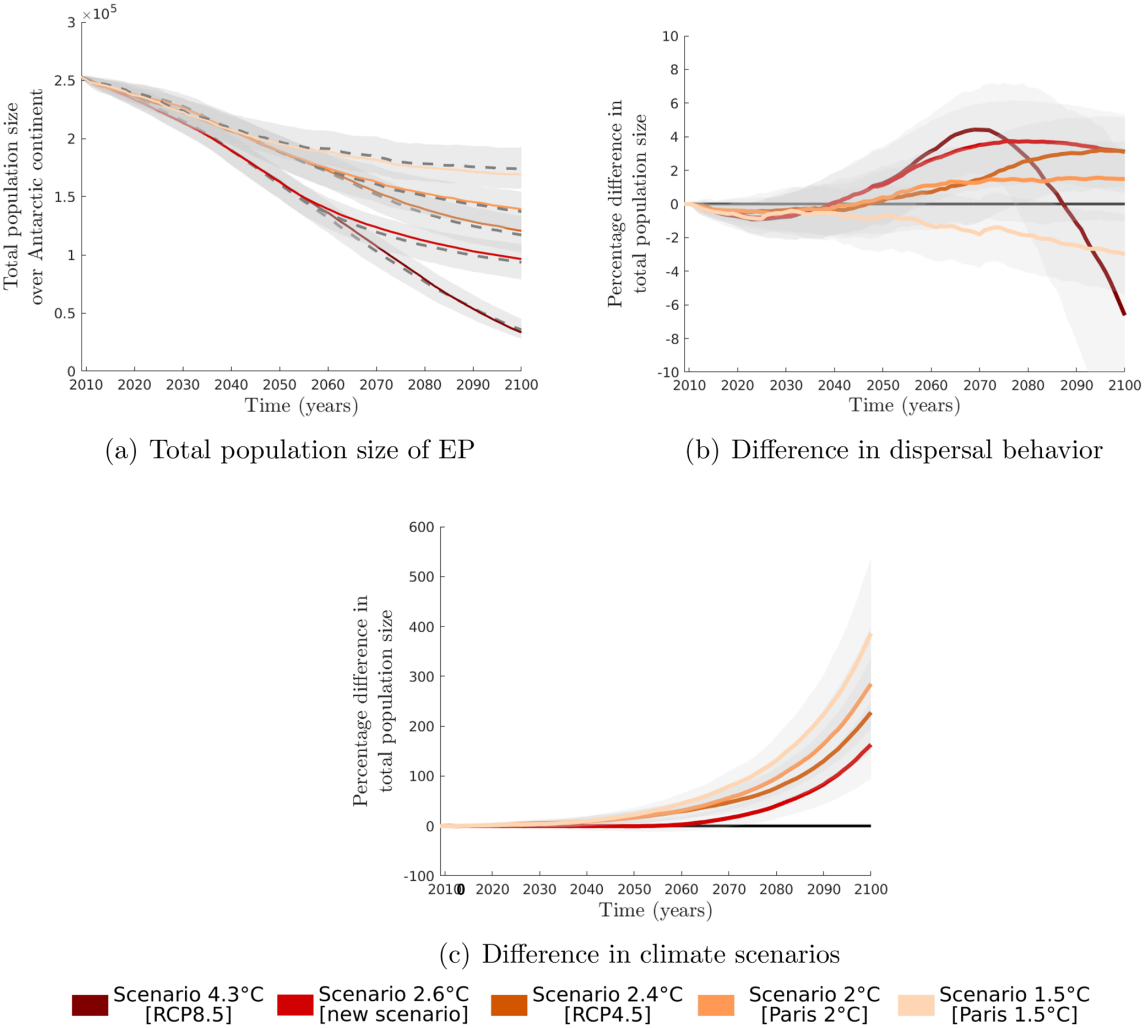
Projection of the total population size of the emperor penguin from 2009 to 2100 using the metapopulation demographic model (see defined by (1)) with different climate scenarios. In panel (a), the projected total population size without dispersal (dashed curves) and with semi‐informed dispersal with the most likely parameters provided by our analysis (plain curves); the gray regions correspond to the confidence intervals 1% around the median. In panel (b), we present the percentage difference of population size between the projection with a semi‐informed dispersal and the projection without dispersal for each climate scenario. In panel (c), we present the percentage difference of population size between the projection with the worst climate scenario, 4.3°C [RCP8.5] and the other scenarios.

According to our predictions, the emperor penguins are most likely to disperse through semi‐informed dispersal with a small mean distance of 414 km and small emigration rates. This dispersal process is estimated to result in a greater global population, up to 7%, compared to a scenario without dispersion when climate scenarios lead to significant population declines (from scenario 4.3°C and scenario 2°C, as shown in Figure 5). However, under a climate scenario of 1.5°C [Paris 1.5°C], which causes lesser declines, our newly estimated dispersal processes do not improve the global population size compared to a scenario without dispersion, but may actually reduce it. Ultimately, the impact of dispersal on future global population size is expected to be relatively small compared to the effects of climate change mitigation (Jenouvrier et al. 2021, 2020) (Figure 5).

## 4 Discussion

Using an innovative mechanistic‐statistical model that integrates empirical demographic and genetic data, we (1) statistically inferred the most likely dispersal behavior used by emperor penguins to move between colonies across Antarctica, (2) mathematically quantified the mean dispersal distance at which individuals are predicted to settle in a new colony, and (3) estimated average emigration rates per colony. While individual dispersal events are not directly observed, the integration of real‐world genetic and demographic data allows us to infer plausible patterns of dispersion. Our model predicts that emperor penguins are more likely to leave colonies experiencing unfavorable conditions and relocate randomly among other colonies. Although average emigration rates remain low, large‐scale dispersal events may still occur locally under specific conditions. Inferred dispersal distances suggest movements over relatively short ranges. We discuss these novel estimations of dispersal processes in the context of potential individual movements documented via satellite telemetry tags and colony movements captured via VHR satellite imagery. We note that neither satellite telemetry tags nor VHR satellite imagery allow direct characterization of dispersal rates, distances, and behaviors but are the only information available to date to speculate about dispersal processes. Unraveling those dispersal processes will also reduce uncertainties in future population projections of emperor penguins necessary for ongoing conservation and management actions. By incorporating this new understanding of dispersion mechanisms into projection models (Jenouvrier et al. 2017), the prediction of the global population trend for emperor penguins, under different climate scenarios, reveals that the influence of dispersion is minimal compared to the influence of climate change mitigation.

### 4.1 New Likelihood Function

The accuracy of inferring how landscape affects dispersal depends on how different types of habitats or geographical elements influence movement (Jaquiéry et al. 2011). Classical genetic methods based on simple dispersal assumptions (e.g., Euclidean distances or least‐cost distances approaches (Wright 1943; Rousset 1997; Broquet et al. 2006)) can provide reliable estimates when the landscape features present strong contrasts in permeability, even with limited sample sizes. However, as the landscape becomes more complex or when different features have similar effects on movement, the power and accuracy of these methods diminish rapidly. In particular, genetic distance‐based methods are more effective at detecting variables that impede dispersal (e.g., barriers) than those that facilitate it, as impeding variables create a stronger signal of reduced gene flow. Consequently, in complex landscapes with diverse environmental structures, combining genetic information with detailed demographic descriptions is crucial to accurately evaluate the impact of landscape variables on dispersal (Jaquiéry et al. 2011). This approach helps mitigate the limitations of genetic methods alone, especially when there are low contrasts between landscape variables, which can obscure the true effects of the landscape on dispersal pathways.

Recently, new mechanistic‐statistical approaches, including reproduction, have been developed to estimate the dispersion of pest species ((Roques et al. 2011) for processionary moths and (Roques et al. 2021) for a bacteria) from genetic data. These approaches have the advantage of remaining relevant even if the degree of differentiation is low or the quantity of observed data is scarce (Roques et al. 2016). However, these approaches do not account for temporal variations or dispersal behaviors, which is particularly important since some species use personal and social information to decide whether to leave a natal or current breeding site and where to settle (e.g., (Doligez et al. 2002)). Such “informed dispersal” behavior (Clobert et al. 2009) enables individuals to settle in better quality habitats, potentially improving their fitness, thus increasing population viability and species persistence, especially in the face of global changes (Ponchon et al. 2015).

Here, we develop a novel likelihood function that incorporates informed departure and settlement behaviors, based on temporal and spatial variations in reproductive strategies and population dynamics. To achieve this level of complexity, the model is conditional on the assumption of a metapopulation being composed of four fixed genetic clusters at Hardy–Weinberg equilibrium, allowing us to focus solely on the evolution of the number of individuals within each cluster. However, we acknowledge that given our representative sampling approach, there could be more than four genetic clusters in the emperor penguin population, and our framework is based on this set of conditions.

Alternatively, for other systems, it may be more appropriate to compute the dynamics of allele frequencies at any time and location, though this approach is more computationally intensive. This dynamic can be modeled by adapting the methods of (Roques et al. 2012), but it requires simulating a system that scales with the total number of alleles in the population—an unfeasible task for our study. Another option is to use multilocus likelihoods for hybrids and backcrosses as developed by (Anderson and Thompson 2002), which involves tracking hybrid dynamics at each time step and computing the corresponding likelihoods.

Therefore, while these alternative approaches offer more flexibility with respect to population structure and do not necessarily assume fixed genetic clusters, they come with their own set of assumptions and greater computational demands. It is important to note that all models, including ours, are simplifications of reality and are conditional on a set of assumptions tailored to the specific goals and limitations of the study. Our chosen approach balances complexity and feasibility, which is particularly suited for the current research context of data‐sparse environments.

Our approach introduces a general, flexible, and efficient mathematical framework for inferring species' dispersal dynamics based on their demographic and genetic structure or dynamics. Beyond estimating dispersal patterns, this approach can also address broader questions in ecology and evolution. For example, recent theoretical developments in evolution suggest that neutral genetic markers can provide insights into genealogy or ancestral lineages among populations under selection (Garnier et al. 2023). Extending our approach to these models could enhance our understanding of the genealogy of long‐lived species, such as the black‐browed albatross, for which obtaining a pedigree is particularly challenging. Genealogical information is crucial for understanding species evolution. Although this approach has been applied to various ecological models, further studies are needed to explore additional questions, apply it to more complex systems, and address the method's known limitations.

### 4.2 Four Genetic Clusters

Due to the species' breeding distribution across harsh and inaccessible areas, complete genetic sampling of all colonies is logistically infeasible. This limitation is not unique to emperor penguins, as restricted sampling is often the only viable option for studying the population genetic structure of many wildlife species. Although additional genetic clusters may exist in unsampled regions, previous studies suggest that geographic distance alone is not a reliable predictor of genetic structure in emperor penguins (Younger et al. 2015) and more broadly Southern Ocean penguins (Cole et al. 2019). For instance, in emperor penguin, Amanda Bay and Pointe Géologie, located 3200 km apart, belong to the same genetic cluster (Younger et al. 2017).

The observed genetic differentiation among the four detected genetic clusters in emperor penguins is likely explained by historical factors rather than contemporary geographic barriers. Previous studies indicate that only three populations of emperor penguins may have survived during the Last Glacial Maximum (LGM), with the Ross Sea acting as a critical refuge (Younger et al. 2015). The LGM has profoundly influenced the genetic structure of many penguin species (Cole et al. 2019). Indeed, consistent genome‐wide signatures of post‐LGM expansion have been detected in penguin species that currently breed south of the LGM sea ice zone, suggesting that many Southern Ocean species retreated to ice‐free refugia during the LGM and rapidly recolonized high‐latitude shores as the ice receded (Cole et al. 2019). These historical refugia have likely shaped the present‐day genetic structure of several penguin species, including emperor penguins (Younger et al. 2015).

We acknowledge that this representative sampling could be seen as a limitation for classical methods, such as BayesAss and coalescent models, which typically require extensive spatial sampling and high genetic differentiation between populations to estimate dispersal rates accurately. Unlike traditional methods that indirectly infer dispersal from genetic structure, our approach explicitly models dispersal dynamics using a combination of a demographic model and a genetic population model. Our demographic model describes the dispersal of all individuals across colonies, while the genetic population model tracks the lineage of individuals over time and assumes that a newborn inherits its genetic cluster from its mother. By integrating these demographic processes and focusing on the explicit movement of individuals, our model overcomes the limitations of traditional genetic methods that rely heavily on genetic differentiation and extensive sampling.

By tracking the movement and lineage of individuals across colonies, our model offers a valuable tool to understand how these populations may respond to ongoing climate change with dispersal behaviors, despite the constraints of limited genetic sampling. This approach sheds light on the complex dispersal dynamics of emperor penguins across Antarctica, contributing to a more comprehensive understanding of their connectivity and resilience in the face of environmental change.

### 4.3 Dispersal Ranges

The posterior distribution of the mean dispersal distance for the best supported model, depicted in Figure 2a, suggests a short dispersal distance relative to the potential distance that tracked juveniles and adults cover after departing the colony (Thiebot et al. 2013; Kooyman et al. 2004). In fact, we found that the most likely dispersal distance of the emperor penguins is around 414 km. Satellite telemetry studies have shown that penguins can cover incredible distances during their searching routes. In the Ross Sea, non‐breeders can travel up to 9000 km (Goetz et al. 2018) to their wintering grounds, and after the molt, adults covered more than 2000 km on their return journey to their colonies (Kooyman et al. 2004). In East Antarctica, one juvenile covered more than 7000 km during the first 8 months after leaving its natal colony in Terre Adélie (Thiebot et al. 2013).

However, the distance covered during the searching phase does not necessarily reflect the dispersal distance. In fact, individual potential dispersal can be reduced by specific behaviors. For example, seabirds exhibit specific behavioral traits, such as a high degree of philopatry (Aebischer and Coulson 1990) and the importance of social cues in the recruitment of new breeders (Reed et al. 1999), which can reduce the dispersal distance of individuals relative to possible movement (Kildaw et al. 2005; Matthiopoulos et al. 2005). For example, the colonies in the Ross Sea are genetically distinct from the rest of the colonies (Younger et al. 2015), suggesting that, despite their large dispersal potential during the non‐breeding season, the dispersal distance of the emperor penguin could be somewhat limited. Additionally, after the demise of Halley Bay, many of the birds of Halley Bay may have relocated to the nearby Dawson‐Lambton colony, while the formation of new colonies elsewhere or movement to other locations of the colony further away is considered less likely (Fretwell and Trathan 2019).

### 4.4 Dispersal Rates

Previous studies have debated the magnitude of emigration rates in emperor penguins, with some studies arguing for large emigration rates (LaRue et al. 2015), while others argue for low emigration rates (Mougin and Van Beveren 1979; Prevost 1961). Although large‐scale emigration events are possible, our research indicates that these occurrences are rare.

Large emigration rates producing massive movements between colonies have been documented in the past two decades from satellite imagery: 1. Some colonies are known to ‘blink’ (disappear in some years, reappear in others) (Fretwell and Trathan 2021); 2. Others are known to relocate to icebergs or ice shelves during late sea ice formation in the autumn (Fretwell et al. 2014); 3. Some colonies have shown dramatic declines, while nearby colonies have increased in size markedly (Fretwell and Trathan 2019). Although blinking, relocation, and massive movement events remain relatively infrequent, they may still have significant impact on local population dynamics or genetic mixing.

Spatially, 17% of the colonies are known to blink. In the past decade, certain colonies experienced intermittent periods of absence. Taking into account this fluctuation over time, the likelihood of a colony being absent in any given year is only 4%. Furthermore, it is anticipated that emigration rates will be low due to the significant number of marked chicks that have been observed to return to Pointe Géologie (Mougin and Van Beveren 1979).

Nonetheless, it is crucial to establish the specific time and space frames in which these rates take place. In this study, we suggest that the overall yearly percentage of emperor penguins dispersing from one breeding site to another is relatively minimal. However, there might be instances of mass emigration occurring sporadically in certain locations (see Figure 3a). This pattern has been noticed in numerous species of seabirds and birds, and it aligns with the philopatric behavior exhibited by these species. As an example, greater flamingos exhibit similar characteristics to emperor penguins in terms of their long lifespan and tendency to breed in one location (philopatric). Generally, they have a low rate of emigration, but when the conditions for breeding are poor at their colony, such as when water levels are low, they relocate together to another breeding location (see (Nager et al. 1996; Balkiz et al. 2010; Johnson and Cézilly 2007)).

In addition, our framework focuses on emigration rates at the population level. However, in many vertebrate species, especially in seabirds, juvenile dispersal is greater than adult dispersal (Clobert et al. 2004). For emperor penguins, massive emigration events are likely to consist mainly of adults in some regions, whereas the low background levels of emigration are likely dominated by juvenile dispersal. Further work should include this age structure in the dispersal demographic model to disentangle the dispersal rates of adults from those of juveniles. However, this would require understanding the detailed mechanisms of density dependence on those two age classes, which are unknown for emperor penguins (Jenouvrier et al. 2012).

Moreover, in cases where emigration and prospecting evolve simultaneously, the emigration strategy that emerges is one in which successful breeders consistently exhibit philopatry, while unsuccessful breeders are more inclined to emigrate, particularly when the breeding success of conspecifics is low (Ponchon et al. 2021). This suggests that large‐scale emigration events in emperor penguins may primarily involve unsuccessful breeders. Future research could incorporate a breeding stage structure into the dispersal demographic model to better understand the dispersal rates of both successful and unsuccessful breeders.

Finally, emigration rates vary substantially among regions. For example, the average annual emigration rate per colony between colonies in the A‐B region is 15.7%. This high rate is likely driven by the lower habitat quality in the A‐B region due to rapid declines in SIC (Abram et al. 2010). The spring season of 2022 saw record low sea ice extent in Antarctica, with the greatest negative anomaly occurring in the central and eastern Bellingshausen Sea, west of the Antarctic Peninsula (Fretwell et al. 2023). Some areas experienced a 100% loss in SIC during November, leading to widespread breeding failure of emperor penguin colonies (Fretwell et al. 2023). These findings suggest that such extreme environmental changes are influencing movement patterns and demographic connectivity within this region.

This elevated emigration rate may suggest a genetically homogeneous population in the A‐B region, potentially representing a new genetic cluster. However, due to the region's inaccessibility and the logistical challenges of sampling, genetic data are scarce, making it difficult to determine whether this population is part of one of the four known genetic clusters or constitutes a distinct cluster. This highlights the need for increased research efforts and enhanced sampling strategies in this understudied region to better understand the genetic structure and demographic connectivity of emperor penguins in the face of rapid environmental change.

### 4.5 Potential Drivers of Dispersal Rates

Zooplankton biomass serves as an indicator of the food sources available to emperor penguins and reflects the dynamics of the lower food web in the Antarctic ecosystem (Offredo and Ridoux 1986; Kirkwood and Robertson 1997; Cherel and Kooyman 1998). Our study revealed that it is the main factor that influences dispersion rates (Figure 4d). Specifically, we observed a negative relationship between zooplankton biomass during the nonbreeding period and the probability of annual emigration, as shown in Figure 4a. Consequently, when resources are abundant before breeding, emperor penguins are less inclined to leave their colony.

Variables related to fast ice also play a significant role in determining the likelihood of emigration. Specifically, the annual probability of emigration is positively influenced by the distance to the nearest fast‐ice edge (Figure 4b). Consequently, emperor penguins are more inclined to leave their colony when it becomes more challenging to access open water. This positive relationship between distance to the nearest fast‐ice edge and emigration probability has also been documented in relation to breeding success (Labrousse et al. 2021).

On the other hand, we observed that demographic factors did not have a significant impact on the average probability of emigration, except for population size (Figure 4d). The larger colonies were found to have a lower average probability of emigration (Figure 4c) and an annual probability of emigration. Despite expectations, smaller, declining, and frequently blinking colonies do not necessarily have higher emigration rates.

Although more work is needed to elucidate the proximate factors of suitable habitat and emigration rates of emperor penguins, our results suggest that massive emigration events occur in habitats with low food availability that cannot sustain large populations and in colonies that are distant from open water.

### 4.6 Dispersal Behaviors and Their Consequences for the Dynamics of the Global Population

Based on the predictions of our new model, emperor penguins tend to migrate from colonies with unfavorable habitats and randomly settle in a new colony, a behavior known as semi‐informed dispersal (Ponchon et al. 2021) (see Table 1).

Previously, (Jenouvrier et al. 2017) have shown that high emigration rates and long‐distance dispersal accelerate the projected global population decline of emperor penguins and decrease the global population size by 65% by 2100 compared to a scenario without dispersal. However, here we show that high emigration rates and long‐distance dispersal are unlikely for emperor penguins. Our model suggested limited dispersal distances and low average emigration rates, leading to a slight increase in the global population compared to a scenario with no dispersal (see Figures 3 and 5a,b in (Jenouvrier et al. 2025)).

Nevertheless, the influence of dispersal behavior, distance, and emigration rate on the future global population size is relatively insignificant compared to the influence of climate change mitigation (Jenouvrier et al. 2020) (see Figure 5c). At the end of the century, there will be no suitable habitat if greenhouse gas emissions continue their current course, resulting in a large decline in the global population, regardless of dispersal processes (Jenouvrier et al. 2020). To mitigate the rapid decline in its worldwide population, it is imperative to limit temperature increases to levels that are considerably below 2°C (Jenouvrier et al. 2021).

## 5 Conclusion

By developing a new likelihood function for an innovative model that integrates genetic information with metapopulation dynamics, we predicted and estimated previously unidentified dispersal patterns in emperor penguins using limited genetic data. The application of this modeling approach has the potential to be used in various species and data‐limited systems to uncover dispersal processes. It has the ability to enhance our understanding of the ranges, speeds, and behaviors of dispersal.

## Author Contributions

**Jimmy Garnier:** conceptualization (equal), formal analysis (equal), methodology (equal), project administration (equal), writing – original draft (lead), writing – review and editing (lead). **Gemma Clucas:** data curation (equal), writing – review and editing (supporting). **Jane Younger:** data curation (equal), writing – review and editing (supporting). **Bilgecan Sen:** formal analysis (equal). **Christophe Barbraud:** data curation (equal). **Michelle LaRue:** data curation (equal). **Alexander D. Fraser:** data curation (equal). **Sara Labrousse:** data curation (equal). **Stéphanie Jenouvrier:** conceptualization (lead), formal analysis (equal), methodology (equal), project administration (equal), resources (equal), supervision (lead), writing – original draft (lead), writing – review and editing (lead).

## Conflicts of Interest

The authors declare no conflicts of interest.

## Data Availability

The SNP data set is available from the Dryad Digital Repository https://doi.org/10.5061/dryad.4s7t3. The codes for the likelihood function and forecasts of emperor penguin population are available from the GitHub repository https://github.com/garnieji/EP_demographic_genetic, and the code for the importance of climatic and demographic covariates is available from the GitHub repository https://github.com/bilgecansen/Emperor_dispersal.
